# Neurobrucellosis Complicating Pregnancy: A Case Report

**DOI:** 10.1155/S1064744994000268

**Published:** 1994

**Authors:** D. Jay Gloeb, Carla Lupi, Mary Jo O'Sullivan

**Affiliations:** 1 Department of Obstetrics and Gynecology Division of Maternal Fetal Medicine University of Miami School of Medicine/Jackson Memorial Medical Center Miami FL USA; 2 Perinatal Associates of Northern California 5301 F Street Suite 110 Sacramento CA 95819 USA

## Abstract

*Background:* Brucellosis in humans is an infectious disease which may occur following contact with infected domestic animals or the ingestion of unpasteurized dairy products. It has rarely been described in pregnancy. The diagnosis, neuropsychiatric manifestations, and management of brucellosis in a 3rd-trimester pregnant woman are discussed.

*Case:* A 24-year-old Mexican female, G_3_P_2002_, at 30 weeks gestation presented with fever, shaking chills, night sweats, a non-productive cough, weakness, nausea, vomiting, anorexia, and vague, dull, upper abdominal pain as well as neuropsychiatric findings. Extensive evaluation revealed serologic and culture evidence of *Brucella* infection. Her worsening neuropsychiatric condition necessitated preterm delivery with satisfactory neonatal and improved maternal outcomes.

*Conclusion:* Bacteriologic infection of pregnant women with neuropsychiatric manifestations should prompt a careful investigation, and brucellosis should be considered if there is a travel history possibly compatible with contact with domestic animals or the ingestion of unpasteurized dairy products.


Infectious Diseases in Obstetrics and Gynecology 1:285–289 (1994)
© 1994 Wiley-Liss, Inc.

### Neurobrucellosis Complicating Pregnancy: A Case Report

**D. Jay Gloeb, Carla Lupi, and Mary Jo O'Sullivan**

*Department of Obstetrics and Gynecology, Division of Maternal Fetal Medicine, University of Miami School of Medicine/Jackson Memorial Medical Center, Miami, FL*

ABSTRACT

***Background:*** **Brucellosis in humans is an infectious disease which may occur following contact with infected domestic animals or the ingestion of unpasteurized dairy products. It has rarely been described in pregnancy. The diagnosis, neuropsychiatric manifestations, and management of brucellosis in a 3rd-trimester pregnant woman are discussed.**

***Case:*** **A 24-year-old Mexican female, $G_3P_{2002}$, at 30 weeks gestation presented with fever, shaking chills, night sweats, a non-productive cough, weakness, nausea, vomiting, anorexia, and vague, dull, upper abdominal pain as well as neuropsychiatric findings. Extensive evaluation revealed serologic and culture evidence of *Brucella* infection. Her worsening neuropsychiatric condition necessitated preterm delivery with satisfactory neonatal and improved maternal outcomes.**

***Conclusion:*** **Bacteriologic infection of pregnant women with neuropsychiatric manifestations should prompt a careful investigation, and brucellosis should be considered if there is a travel history possibly compatible with contact with domestic animals or the ingestion of unpasteurized dairy products.** © 1994 Wiley-Liss, Inc.

KEY WORDS

Antepartum infection, brucellosis, neurobrucellosis

*Brucella* infection is caused by a gram-negative coccobacillus (*Brucella abortus*, *B. melitensis*, and *B. suis*) found primarily in domestic animals such as goats, sheep, cattle, and swine.[1] In humans, it is an acute and chronic infection. Prevalence depends on multiple factors including endemicity and exposure to infected domestic animals and to contaminated dairy products.[2] While there is discussion in the medical literature about the causative role of *Brucella* in human abortion,[1,3–6] brucellosis is rare in pregnancy. Little is known about the psychiatric manifestations and management of protracted *Brucella* infection unresponsive to therapies other than tetracycline.

We report a case of a woman in the 3rd trimester whose acute central nervous system (CNS) symptoms were the first manifestation of brucellosis and whose psychiatric complications were significant enough to warrant preterm delivery to institute definitive tetracycline therapy.

### CASE REPORT

A 24-year-old Mexican female, $G_3P_{2002}$, at 30 weeks gestation presented to the obstetric emergency room at Jackson Memorial Hospital, Miami, FL, complaining of fever, shaking chills, and night sweats for 2 days. She had a non-productive cough, weakness, nausea, vomiting, anorexia, and vague, dull, upper abdominal pain for 3–5 days.

She had had 2 term, uncomplicated spontaneous vaginal deliveries. There was no significant medical or surgical history. The family history was significant for a sister-in-law who died of hepatitis 5 months prior to the patient's admission. The patient

Address correspondence/reprint requests to Dr. D. Jay Gloeb, Perinatal Associates of Northern California, 5301 F Street, Suite 110, Sacramento, CA 95819.

Received September 3, 1993
Accepted April 7, 1994

had immigrated from Mexico City via Texas 1 month prior to being hospitalized. Other than a complaint of mental confusion during the month prior to admission, there was no history of any neurologic or psychiatric condition. The only medications she had been taking were prenatal vitamins and an iron supplement.

On admission, she was alert and oriented. Her blood pressure was 98/62 mm Hg, pulse 88/min and regular, respirations 24/min and regular, and temperature 38.9°C. The relevant physical findings included diaphoresis, pale conjunctivae, and anicteric sclerae. The abdomen was soft with mild tenderness in the right upper quadrant; no palpable hepatosplenomegaly was noted. The fundal height was 27 cm with a normal fetal heart rate. There was no focal neurological finding or palpable lymphadenopathy. The initial laboratory studies revealed a hemoglobin of 8.3 gm/dl, hematocrit 24%, and a white blood cell (WBC) count of 23,000/mm$^3$. There were 48% bands, 29% lymphocytes, 20% polymorphonuclear cells, 2% mononuclear cells, and 1% basophils. The platelet count was 145,000/mm$^3$ and the sickle cell preparation was negative. The serum $B_{12}$ was 732 pg/ml and the serum folate was 4.2 pg/ml. The serum sodium was 128 meq/l, potassium 2.9 meq/l, chloride 94 meq/l, carbon dioxide 17 meq/l, glucose 91 mg/dl, blood urea nitrogen 3 mg/dl, and creatinine 0.4 mg/dl. The bilirubin and liver enzymes were within normal limits. A urinalysis revealed moderate ketones. A chest X-ray demonstrated mild perihilar and peribronchial thickening. She was given intravenous (IV) fluids to bring her electrolyte levels to normal.

Following admission, she was noted to have mild hepatosplenomegaly on abdominal ultrasonography, and hyperplasia and megaloblastic changes were noted on a bone marrow biopsy performed because of leukopenia. Special stains and cultures for acid-fast bacilli and fungi were negative. Skin tests for mumps, Candida, and tuberculosis were negative. Rheumatoid factor, antinuclear antibody test, and serum rapid plasma reagin test were all negative; C3 was 102 mg/dl and C4 was 26 mg/dl (normal, 3rd trimester: C3 165 ± 4, C4 37 ± 2). Induced sputum was negative on both direct and concentrated smears for acid-fast bacilli.

On the 3rd hospital day, she became delirious, was oriented to person only, and had gross hand tremors. Her blood pressure was 130/70 mm Hg, pulse 128/min, and respirations 28/min. The fetal heart tones were 184/min. Her temperature remained elevated with a maximum reading of 40.1°C. Arterial blood gases on room air revealed a mild respiratory alkalosis with normal oxygen saturation. The delirium persisted. She became combative, disoriented, and uncooperative. Her physical examination was unchanged. A computerized tomographic study of the brain with and without contrast was normal.

A lumbar puncture revealed no significant abnormalities with normal protein electrophoretic studies. Gram's, acid-fast bacilli, and India ink stains, cryptococcal antigen, and cerebrospinal fluid (CSF) culture were all negative. The CSF Venereal Disease Research Laboratory test was non-reactive. Blood studies for acute and chronic hepatitis A and B, mononucleosis, and human immunodeficiency virus as well as thin and thick smears for malaria were all negative. Urine and multiple blood cultures as well as stool for ova and parasites were all negative.

Since no studies were diagnostic, she was empirically started on IV ampicillin and trimethoprim-sulfamethoxazole for possible typhoid fever or listeriosis. Peripheral hyperalimentation was started because of reduced food intake and intermittent nausea and vomiting. Additional serum was collected for herpesvirus, arboviruses, and leptospirosis The herpesvirus titer was found to be 1:32, while titers for Eastern, St. Louis, and Denque viruses and leptospirosis were all negative. A subsequent herpesvirus titer measured 1:64 and all other convalescent titers were negative.

After 1 day of antibiotic therapy, she became afebrile, but continued to experience periods of confusion, lethargy, agitation, combativeness, depression with paranoid features, and intermittent auditory hallucinations through hospital day 7.

On hospital day 8 (4th day of antibiotic therapy), the fever recurred. A febrile agglutinin panel revealed a serum *Brucella* titer of 1:320. The IV ampicillin was discontinued and the IV trimethoprim-sulfamethoxazole dose was increased based on the trimethoprim component, i.e., 400–500 mg/day (10–15 mg/kg/day in divided doses).[7]

A repeat serum *Brucella* titer 6 days later (hospital day 14) was 1:640. A blood culture previously obtained on hospital day 4 grew *Brucella*, species unidentified.

After 12 days of IV trimethoprim-sulfamethoxazole, she remained agitated with persistent gross tremors of the hands, continually paced the room, and was noted to be experiencing auditory hallucinations and occasional confusion. An electroencephalogram performed on hospital day 23 was negative. Her altered mental status continued, waxing and waning in severity despite multiple antipsychotic agents including haloperidol, thioridazine hydrochloride, mesoridazine besylate, and chlorpromazine hydrochloride. A repeat computerized tomographic study of the brain and lumbar puncture was normal.

It became obvious that the patient's persistent and significant neuropsychiatric manifestations, now felt to be secondary to *Brucella* infection, were not responding to the IV trimethoprim-sulfamethoxazole. All fetal surveillance studies were normal with good interval growth. Since she was not improving and tetracycline would be more definitive therapy, an amniocentesis for lecithin/sphingomyelin ratio was performed at 34 weeks gestation (hospital day 27). The ratio was 1.9; therefore, 1 week later, following an induction, she spontaneously delivered a 2,950 g female neonate with Apgar scores of 7, 8, and 9 at 1, 5, and 10 min, respectively. The patient was immediately started on tetracycline, 500 mg q 6 h.

### NEUROLOGICAL ASSESSMENT

A neurological assessment was conducted prior to delivery after the neuroleptic agents and IV trimethoprim-sulfamethoxazole had been discontinued for 3 days. She was alert and oriented. Her speech was fluent, and repetition and naming were intact. Calculations were performed correctly, as were right and left discriminatory tasks. Short-term memory tasks at 5 min revealed a recall of 1 of 3 objects. Long-term memory was poor. A cranial nerve examination was remarkable for bilateral horizontal end-gaze and vertical nystagmoid movements. Normal optokinetic movements were present. A motor examination was significant for diffuse marked increased tone with cogwheel rigidity at the wrists. Bilateral symmetric hyperreflexia with bilateral ankle clonus and a brisk jaw-jerk were present. Strength in all muscle groups was present. Bradykinesia was prominent. Frontal-release signs, snout, grasp, and palmar-mental reflexes were present. Coordination was intact with normal finger-to-nose, heel-to-shin, and rapid alternating movements. The patient's gait was remarkable for a stooped posture and retropulsion. Some of these findings were extrapyramidal signs attributed to the neuroleptic therapy.

On postpartum day 4 (day 42 of hospitalization and 4th day of tetracycline therapy), her mental status was remarkable for a muted affect and evidence of depression, though her concentration and memory were improved. She denied auditory hallucinations or paranoid delusions. A cranial nerve examination was significant for ocular ataxia and a tremulous tongue. Motor testing revealed persistent bradykinesia, cogwheel rigidity, and a festinating gait.

On hospital day 45, the patient was transferred to the neurology service where all the neuroleptics were discontinued. On hospital day 50, her behavior was still bizarre and combative with unexpected outbursts. Chlorpromazine hydrochloride, 10 mg, was restarted and she again partially responded. She was discharged home 2 days later. On that day, assessment on the Mattis Dementia Scale and the Boston Naming Scale (word retrieval) showed mild and moderate impairment, respectively. Her discharge medications included chlorpromazine hydrochloride, 25 mg bid; diphenhydramine hydrochloride, 25 mg bid; benztropine mesylate, 2 mg q 8 h prn; tetracycline, 500 mg q 6 h; multivitamins; and an iron supplement. A follow-up neurologic examination 2 weeks after discharge revealed only increased deep-tendon reflexes.

### DISCUSSION

Brucellosis can involve the CNS in a variety of forms including meningoencephalitis, subarachnoid hemorrhage, meningomyelitis, and neuritis.[8,9]

Initially, our patient demonstrated many of the systemic manifestations of classic acute *Brucella* infection[10] including fever, chills, sweating, generalized weakness, anorexia, and a non-productive cough. *Brucella* endotoxin "contributes significantly to the pathogenesis of illness in brucellosis."[11] Leukopenia, anemia, thrombocytopenia, mildly elevated liver function tests and total and direct bilirubin, fever, abdominal tenderness, splenomegaly, and bone marrow granulomas are all found with varying frequency in the acute phase of brucellosis.[10]

In our case, in addition to serologic evidence, acute *Brucella* was conclusively diagnosed by culture, though it was never full speciated. The history of eating unpasteurized goat's cheese in Mexico (previously reported as a cause of brucellosis[12]) elicited from the patient after she delivered as well as her marked clinical response to the appropriate antibiotic in the absence of any previous mental condition was corroborating evidence of neurobrucellosis. Effective standard antibiotic therapy for acute or subacute brucellosis in the past has been a combination of tetracycline and streptomycin.[7,10,13] Presently, the preferred chemotherapy in adults is doxycycline and rifampin.[7]

It was unclear whether the patient's condition represented CNS involvement (primary neurobrucellosis) or the toxic systemic effects of *Brucella* endotoxin (secondary neurobrucellosis). Her altered mental status may have been partially worsened by the neuroleptics she received. Her cranial nerve, motor, reflex, and gait dysfunction may have been secondary to the antipsychotic agents causing a parkinsonian syndrome and akathisia[14] and, finally, the CNS reacts to sulfonamides causing headache, depression, and hallucinations.[15]

Neuropsychiatric symptoms occur in approximately two-thirds of those with brucellosis, whereas in less than 10% evidence of CNS *Brucella* invasion can be found.[10] Spinal fluid findings consistent with brucellosis include pleocytosis, elevated protein, and increased immunoglobulin concentration. The symptoms are attributed to the irritable effects of *Brucella* toxins on the nervous system.[9] Neuropsychiatric symptoms include delusions of persecution, psychosis, depression, and irritability.[16,17]

In the non-pregnant patient, CNS brucellosis has a variable onset relative to the time of infection.[8] There is a wide spectrum of clinical disease from acute to chronic with minimal, local disease, or massive, widespread CNS disease. Brucellosis may be progressive, episodic, or transient. Neurological complications can occur at the beginning of the disease, but often occur after a few febrile episodes, during convalescence, or long after the illness.[8] In pregnancy, effective intervention with tetracycline must be weighed against the risks to the fetus of bone growth retardation, dental enamel staining and hypoplasia, and maternal hepatic and pancreatic dysfunction.[18–20] While rare, neurobrucellosis should be considered in the differential diagnoses of neurological disorders occurring inpregnancy. This case is unique because the neuropsychiatric manifestations of the infection prompted preterm delivery for definitive antibiotic therapy and, fortunately, in the 3rd trimester when fetal pulmonary maturity was possible. Under less favorable circumstances, the risks of tetracycline and the failure of alternative therapy would have to be weighed against the risks of severe, prolonged maternal neurologic and psychiatric deterioration.

### REFERENCES

1. Sarram M, Feiz J, Foruzandeh M, Gazanfarpour P: Intrauterine fetal infection with *Brucella melitensis* as a possible cause of second-trimester abortion. Am J Obstet Gynecol 119:657–660, 1974.
2. How does *Brucella abortus* infect human beings? Lancet 2:1180, 1983.
3. Fernihough TJ, Munoz WP, Mahadeyo I: The role of *Brucella abortus* in spontaneous abortion among the black population. S Afr Med J 68:379–380, 1985.
4. Porreco RP, Haverkamp AD: Brucellosis in pregnancy. Obstet Gynecol 44:597–602, 1974.
5. Veiga G: Brucelose no aparelho genital feminino. Matern Infanc (Sao Paolo) 33:235–270, 1974.
6. Goobar J, Blank B: La brucelosis cronica en los problemas gineco-obstetricos. Obstet Ginecol Lat Am 22:283–294, 1964.
7. Hall WH: Modern chemotherapy for brucellosis in humans. Rev Infect Dis 12:1060–1099, 1990.
8. Fincham RW, Sahs AL, Joynt RJ: Protean manifestations of nervous system brucellosis. Case histories of a wide variety of clinical forms. JAMA 184:97–103, 1963.
9. Abramsky O: Neurological features as presenting manifestations of brucellosis. Eur Neurol 15:281–284, 1977.
10. Spink WW: The Nature of Brucellosis. Minneapolis: University of Minnesota Press, 1956.
11. Abernathy RS, Spink WW: Studies with brucella endotoxin in humans: The significance of susceptibility to endotoxin in the pathogenesis of brucellosis. J Clin Invest 37:219–231, 1958.
12. Eckman MR: Brucellosis linked to Mexican cheese. JAMA 232:636–637, 1975.
13. Spink WW: Current status of therapy of brucellosis in human beings. JAMA 172:697–698, 1960.
14. Baldessarini RJ: Drugs and the treatment of psychiatric disorders. In Goodman Gilman A, Goodman LS, Rall TW, Murad F (eds): The Pharmacological Basis of Therapeutics. 7th Ed. New York: Macmillan, pp 402–408, 1985.
15. Mandell GL, Sande MA: Animicrobial agents. In Goodman Gilman A, Goodman LS, Rall TW, Murad F (eds): The Pharmacological Basis of Therapeutics. 7th Ed. New York: Macmillan, p 1106, 1985.
16. Hobbs FB: A case of *Brucella abortus* infection. Lancet 2:683–685, 1931.
17. Leys DG: Abortus fever. Br Med J 1:187–189, 1943.

18. Whalley PJ, Adams RH, Combes B: Tetracycline toxicity in pregnancy. Liver and pancreatic dysfunction. JAMA 189:357–362, 1964.
19. Demers P, Fraser D, Goldbloom RB, et al.: Effects of tetracyclines on skeletal growth and dentition: A report by the Nutrition Committee of the Canadian Paediatric Society. Can Med Assoc J 99:849–854, 1968.
20. Brearley LJ, Stragis AA, Storey E: Tetracycline-induced tooth changes. I. Prevalence in pre-school children. Med J Aust 55:653–658, 1968.
